# Plasma Concentration of Advanced Glycation End-Products From Wild Canids and Domestic Dogs Does Not Change With Age or Across Body Masses

**DOI:** 10.3389/fvets.2021.637132

**Published:** 2021-01-26

**Authors:** Ana Gabriela Jimenez

**Affiliations:** Department of Biology, Colgate University, Hamilton, NY, United States

**Keywords:** wild canid, domestic dog (*Canis familiaris*), body mass, age, advanced glycation end product

## Abstract

Dogs provide a physiological paradox: In domestic dogs, small breeds live longer lives than large breed dogs. Comparatively, a wild canid can be a similar size than many large breed dogs and outlive their domestic cousin. We have previously shown that oxidative stress patterns between domestic and wild canids differ, so that wild canids invest in a robust antioxidant system across their lives; whereas domestic dogs tend to accumulate lipid damage with age. There is a close association between oxidative stress and the production of a carbohydrate based-damage, Advanced Glycation End-products (AGEs). AGEs can bind to their receptor (RAGE), which can lead to increases in reactive oxygen species (ROS) production, and decreases in antioxidant capacity. Here, I used plasma from wild and domestic canids to address whether blood plasma AGE-BSA concentration associated with body mass and age in domestic dogs; And whether AGE-BSA concentration patterns in blood plasma from wild canids are similar to those found in domestic dogs. I found no correlation between circulating AGE-BSA concentration and body size or age in either domestic dogs and wild canids. These data suggest that AGEs formation may be a conserved trait across the evolution of domesticated dogs from wild ancestors, in opposition to oxidative stress patterns between these two groups. And, that, in domestic dogs, lipid metabolism, rather than carbohydrate metabolism, may be upregulated to yield the previously found differences in circulating lipid damage across lifespan and body sizes.

## Introduction

The domestic dog is one of the most morphologically, and phenotypically diverse mammals known, with body sizes expanding from a Chihuahua to a Great Dane within a single species (1). This broad range in body sizes seems to be dictated in part by a single Insulin growth factor-1 (*IGF1*) haplotype (2), which may also be correlated to a serum IGF-1 reduction in small breed dogs compared with large breed dogs (3). Across body sizes, small breed dogs live significantly longer than large breed dogs [(1) and references therein], unlike patterns often seen across mammalian species of differing body sizes. However, the underlying physiological mechanisms that may explain general aging differences across size classes of domestic dogs are varied, and include differences from tissue- and cell-levels (1, 4, 5). In comparison, wild canids that face natural selection rather than rigid artificial selection practices, are known to have large body sizes and live longer lives compared with large breed dogs (6).

At the cell-level, oxidative stress has been one process that has gained momentum as a physiological mechanism under selection so that small and large animals may be able to promote different aging rates (7). Oxidative stress is a balance, inherent to all aerobic organisms, between the potential damage that could be accrued by reactive oxygen species (ROS) and the resources cells have to thwart that damage through the antioxidant system (4). Using plasma and red blood cells (RBCs) from domestic dogs and wild canids of different ages and body sizes, our lab found that wild and domestic dogs exhibit different patterns in components of oxidative stress which may contribute to their different patterns in aging with respect to body size. We found that lipid damage increases with age in domestic dogs. In contrast, total antioxidant capacity (TAC) increases with age in wild canids and TAC and, antioxidant enzymatic activity, glutathione peroxidase (GPx), increase as a function of age/maximum lifespan (MLSP) in wild canids. Surprisingly, we found that small breed domestic dogs have significantly higher circulating lipid damage on average compared with large breed dogs (6), though it remains unclear whether this accumulation is due to increased rates of lipid damage or decreased rates in clearing said damage. These data suggest that artificial selection in domestic dogs may have selected for decreased antioxidant capacity, and that small breed dogs may be “surviving” with increased oxidative damage (6). These findings warrant the empirical consideration of other damage-producing metabolic pathways that may be associated with oxidative stress and aging.

As metabolites of glucose react with amino acids of proteins and lipids through Maillard reactions an irreversible by-product, known as advanced glycation end-product (AGE) is formed as end stage of carbohydrate metabolism (8). First, non-enzymatic glycation of a protein results in an Amadori rearrangement which is a reversible reaction (8). However, the Amadori product reaches equilibrium over weeks, but can still undergo poorly defined rearrangements and irreversibly form AGEs (9). AGEs can accumulate with age particularly in long-lived proteins such as collagens and crystallins and their formation, especially when it enters the circulation, renders irreversible damage to all biological macromolecules (8, 10). The Maillard theory of aging proposes that the slow and continuous accumulation of AGEs may be a factor in aging rates (10). The majority of AGEs that accumulate with age are glycoxidation products, formed by glycation and oxidation reactions from glucose or ascorbate (11). Through a complex series of reactions, oxidative stress may be involved in AGE formation and AGEs may, in turn, induce oxidative stress (8, 12). AGEs can exert their cellular function via interactions with their receptor (RAGE). Members of the S100/calgranulin family are ligands of RAGE and RAGE within the family of receptors for lysophosphatidic acid (LPA) (13, 14). Activation of RAGE can lead to the production of ROS (8, 12). Additionally, reactive carbonyl species (RCS) formed by oxidation of carbohydrates, lipids, and amino acids have been identified as intermediates for the irreversible formation of AGEs (15). Both glucose, its oxidation products and Amadori products can generate free radicals (16), and perpetuate further oxidative damage. An excess of AGEs in blood is a characteristic of diabetes, atherosclerosis, and is associated with most age-related chronic diseases, including those derived from inflammatory pathways (8, 10, 17, 18).

Thus, because of their close association with oxidative stress pathways, and their potential link with aging rates, a quantification of AGEs in domestic dogs and wild canids may elucidate physiological mechanisms in the aging process of these species. Here, I address two questions: (1) how does blood plasma AGE-BSA concentration associate with body mass and age in domestic dogs? and (2) Are AGE-BSA concentration patterns in blood plasma from wild canids similar to those found in domestic dogs? This approach provides evolutionary data on potential aging mechanisms utilized in this group of mammals and allows for elucidation of physiological patterns that may be associated with domestication and artificial selection.

## Materials and Methods

### Collection of Blood Samples

Blood samples of domestic and wild canids were collected from zoos and veterinarians, as blood serves as a reservoir of metabolic by-products, including AGEs. Only healthy, not actively reproducing individuals of known age were included. None of the dogs included in this study were taking any metabolic, neurological, or endocrine medications and none were obese. Blood samples were collected, spun to separate plasma from red blood cells (RBCs), and frozen immediately. Samples were transported to our lab at Colgate University on dry ice and stored at −80°C until further use. For each individual, we collected information regarding body mass, sex, and age at blood draw. Domestic dog samples included individuals of any age. Domestic dogs were categorized into three size classes based on their body weight: small (up to ~10 kg), medium (~10–20 kg) and large (~20 kg and up) (1). Sample sizes for each species are listed in Table 1. Diet of each animal was not controlled for in this study. Obtaining blood samples was done under the guidelines of the Colgate University Institutional Care and Use Committee.

**Table 1:** Samples sizes of each wild canid specie and domestic dog sizes.

**Species**	***N***
African wild dog	3
Arctic fox	1
Bushdog	1
Coyote	26
Dhole	1
Domestic dog, large breeds	74
Domestic dog, medium breeds	13
Domestic dog, small breeds	46
Gray wolf	1
Maned wolf	6
New guinea singing dog	1

### Advanced Glycation End-Product Competitive ELISA

An Advanced glycation end-product (AGE-BSA) competitive ELISA kit (cell biolabs cat no. STA-817) was used. Fifty microliter of plasma from each individual per species was plated in duplicate and an estimation of the concentration of AGE-BSA in plasma samples based on an AGE-BSA standard curve was performed, as described by the manufacturer. Plates were read at 450 nm using a TECAN infinite m200 plate reader. Each individual's AGEs-BSA concentration can be found in Supplementary Table 1.

### Statistics

AGE-BSA data were analyzed using linear regressions as a function of body size and age, first. Additionally, I used linear regressions to correlate the ratio between age and maximum lifespan (MLSP) (6). I obtained lifespans of each American Kennel Club (AKC) recognized breed from the AKC website, and maximum lifespan of wild canids from Anage (19, 20). Using these numbers, I estimated a ratio of age/MLSP per species and AKC recognized breed. This ratio provides a more accurate estimation of where each individual is in relative to the lifespan of each species and thus provides a measure of age scaled by species longevity. Mixed domestic dog breeds were not included in age/MLSP correlations. Additionally, I used an ANOVA to test differences in AGE-BSA across different domestic dog sizes.

## Results

### Linear Regressions

**Figure 1:**
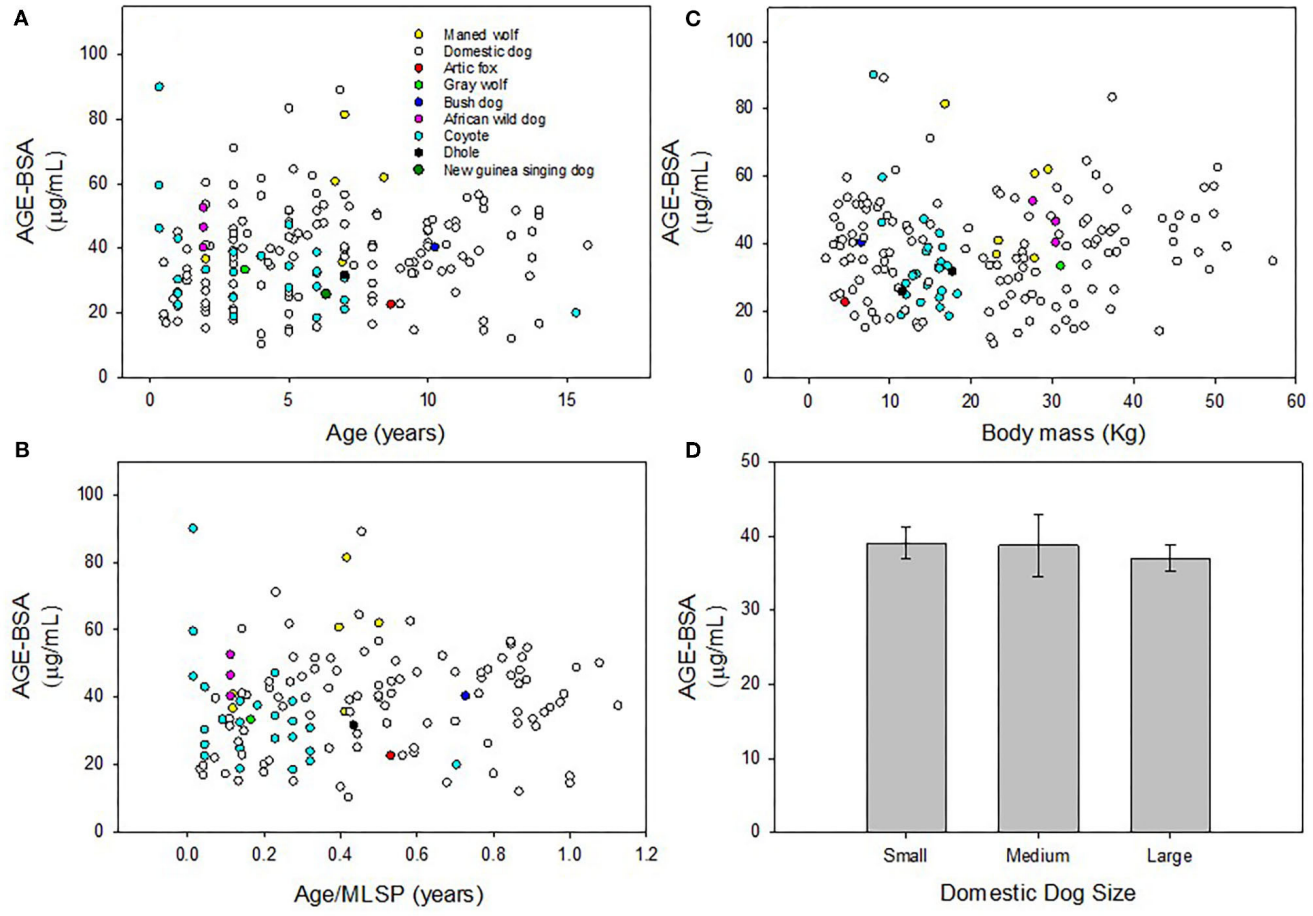
**(A)** Plasma concentration of AGE-BSA in domestic dogs and wild canids were not significantly correlated with age, respectively (*p* = 0.12; *p* = 0.19). **(B)** There was no correlation between AGE-BSA and age/MLSP in domestic or wild canids, respectively (*p* = 0.16; *p* = 0.56). **(C)** There were no correlations between AGE-BSA and body mass in domestic or wild canids, respectively (*p* = 0.53; *p* = 0.28). **(D)** There were no differences in AGE-BSA concentration across three size classes of domestic dogs (*F* = 0.300; *p* = 0.74). Non-significant linear regressions were not plotted. Results were considered significant when p < 0.05.

Plasma concentration of AGE-BSA in domestic dogs were not significantly correlated with age (y = 0.51x + 34.64; *r*^2^ = 0.018; *p* = 0.12; Figure 1A), age/MLSP (y = 7.23x + 34.42; *r*^2^ = 0.022; *p* = 0.16; Figure 1B) or body mass (y = 0.056x + 36.68; *r*^2^ = 0.003; *p* = 0.53; Figure 1C). Plasma concentration of AGE-BSA in wild canids were not significantly correlated with age (y = −1.048x + 42.12; *r*^2^ = 0.044; *p* = 0.19; Figure 1A), age/MLSP (y = −8.60x + 39.64; *r*^2^ = 0.0093; *p* = 0.56; Figure 1B), or body mass (y = 0.41x + 30.45; *r*^2^ = 0.031; *p* = 0.28; Figure 1C).

### ANOVA

There were no differences across three size classes of domestic dogs in AGE-BSA concentration in blood plasma (*F* = 0.30; *p* = 0.74; Figure 1D).

## Discussion

I found that blood plasma AGE-BSA concentration shows no correlation with body mass or age in domestic dogs, and that wild canids show a similar pattern, in opposition to previous data collected in oxidative stress in domestic and wild canids. AGEs are known to accumulate readily in long-lived proteins such as collagen or crystallin proteins in the eye (21). Skin samples were obtained from a variety of mammalian species with varying lifespans and of different ages. Pentosidine, a specific AGE, formation rates in skin collagen showed increases as a function of age in different mammalian species, but it especially correlated to faster-lived species such as the rat and the shrew compared to slower-lived species like monkeys and humans (21). In opposition, long-lived breeder fukomys mole-rats accumulate significantly higher total AGE and its derivatives compared with shorter-lived non-breeders of the same species (22). These studies demonstrate that the accumulation of AGEs with age may not be a direct correlation. In dogs, we know that repeated measured of glycosylated hemoglobin in beagles demonstrated no significant variation over time (23), however, glycated hemoglobin is diabetic dogs is significantly higher than normal dogs (24), demonstrating that this variable may not change unless pathological conditions arise. Additionally, AGEs bind to their multiligand receptor for AGEs (RAGE), a member of the immunoglobin family, without acceleration or clearance of AGEs, but instead with an activation of pathways linked to inflammation, among others (25). Thus, AGEs may have implications to oxidative stress and inflammatory pathways.

Some AGEs and their precursors are implicated as sources of oxidative stress, so that they may accelerate oxidative damage during aging (8). The association between RAGE and AGEs can trigger rapid generation of ROS, which may lead to decreasing antioxidant enzymes as in the case of superoxide dismutase (SOD), catalase (CAT) activities and reducing glutathione stores (17, 25, 26). The free radicals generated by glycoxidation reactions and cellular metabolism, can cause lipid peroxidation. The transformation of Amadori products into AGEs forms reactive dicarbonyl species, which leads to glycative stress. These dicarbonyls can further propagate and accentuate oxidative stress (26). The formation of AGEs may be aerobic or anaerobic, while the formation of Advanced Lipid End-products (ALEs), like the metabolism of lipids requires oxidative chemistry to form intermediates (15). ALEs come in the form of MDA, which is a product of lipid peroxidation (15). Here, I found no correlation with AGE-BSA concentration in plasma and age, age/MLSP, and body size in domestic dogs. We have previously shown circulating lipid damage increases in domestic dogs as they age, and the increases in circulating lipid damage in small breeds compared with large breeds (6). We also know that, at least in primary fibroblast cells isolated from small and large breed dogs, membrane fatty acid composition is not different (27), so that increases in lipid peroxidation rates in domestic dogs may not be stemming from inherent lipid composition differences in their cellular membranes. Taken together, these data imply that AGEs concentrations may be kept at low concentration in domestic dogs across their lifespan, unless pathological states develop; And, that lipid metabolism, rather than carbohydrate metabolism, may be upregulated in domestic dogs to yield the previously found differences in circulating lipid damage across lifespan and body sizes (6) or that any damage associated with AGEs is accumulated through lipid damage rather than glycative stress.

The association between RAGE and AGEs can also trigger an upregulation of inflammatory pathways. Thus, an accumulation of AGEs may influence inflammation patterns and increase “inflammaging,” which is defined as an increase in markers of inflammatory activity that occurs as mammals age (10, 17, 25, 26, 28). Mechanistically, inflammaging seems to be the consequence of cumulative lifetime exposure to antigenic load, or an accumulation of damage, such as that produced by AGEs (17, 29). Inflammation due to aging can be caused by accumulation of pro-inflammatory tissue damage, accumulation of pathogens due to an ill functioning immune system, dysfunctional host cells, to name a few (30). Immunosenescence is well-documented in dogs (31, 32), however, only a handful of studies have addressed pro-inflammatory pathways in older dogs (31). Using primary fibroblast cells from young and old individuals from small and large dog breeds, we found that large breed puppies have significantly less background interleukin (IL)-6, a pro-inflammatory cytokine, concentrations compared with small breed puppies (Jimenez et al., in review). The mechanism behind this pattern has yet to be fully elucidated, but others have found that decreased mitochondrial membrane uncoupling found in large breed dogs' primary fibroblast cells, coupled with lower potential of β-oxidation and accumulation of acylcarnitines can promote inflammation (5), which follows with the fact that our work suggests that large breed dogs seem to have a higher background IL-6 concentration (Jimenez et al., in review). Together with the data presented in the current study, it can be suggested that increases in pro-inflammatory cytokines may not be related to interactions between AGE and RAGE in domestic dogs.

Circulating serum AGEs is a careful balance between endogenous and exogenous production of these molecules, and their clearance through enzyme reactions, receptor relationships, and renal excretion (33). In humans, normal renal function leads to a rapid clearing of AGEs, whereas renal malfunction or diabetic patients with renal complications demonstrate increase in AGEs in tissues (34). A similar result was found in dogs, where AGEs levels were higher in diabetic dogs compared with normal controls (35). However, higher AGE concentration and renal failure were not correlated suggesting that clearance rates in dogs are not the main reason for AGEs accumulation, and rather AGEs formation or production would be the main mechanism for accumulation (35). That there are similar correlations between AGE-BSA in domestic dogs and wild canids is unexpected, especially when considering that we found no correlation between circulating lipid damage with age, or body size in wild canids, however, this may be due to an increase in TAC and antioxidant enzymes in wild canids compared with domestic dogs (6). These data may suggest that clearance rates in wild canids may be similar to those of domestic dogs, and that AGEs formation is a conserved trait across the evolution of domesticated dogs from wild ancestors. It should also be noted that domestic dogs and zoo-kept wild canids are likely to have significantly different diets that could have an effect on circulating AGEs, however, comparisons between home-made (raw) and dry diets in the urine of domestic dogs showed that dogs eating a home-made diet had significantly lower AGEs compared to those eating a dry diet (36), which is not seen in our comparison.

## Conclusions

Aging renders many animals insulin-insensitive and intolerant to glucose, a situation where AGEs generation may accrue (17). However, using plasma samples from wild canids and domestic dogs, here, I found no correlation between AGE-BSA concentration and body size or age, implying that clearing rates for this type of damage are sufficient in this group of animals and may not accumulate until pathological conditions, such a diabetes, are reached. It should be noted, however, that samples collected from wild canids were considerably lower than those collected from domestic dogs, which may have limited or obfuscated some of the generalized patterns of these data.

## Data Availability Statement

The original contributions presented in the study are included in the article/Supplementary Material, further inquiries can be directed to the corresponding author/s.

## Ethics Statement

The animal study was reviewed and approved by Colgate University Institutional Care and Use Committee. Written informed consent for participation was not obtained from the owners because we used blood from tests that were already happening, and owners were asked verbally to participate.

## Author Contributions

AJ collected samples and data, analyzed data, wrote drafts of this manuscript, and secured funding for this paper.

## Conflict of Interest

The author declares that the research was conducted in the absence of any commercial or financial relationships that could be construed as a potential conflict of interest.
